# Developing sustainable software solutions for bioinformatics by the “ *Butterfly*” paradigm

**DOI:** 10.12688/f1000research.3681.2

**Published:** 2014-08-01

**Authors:** Zeeshan Ahmed, Saman Zeeshan, Thomas Dandekar

**Affiliations:** 1 Department of Neurobiology and Genetics, Biocenter, University of Wuerzburg, Wuerzburg, 97074, Germany; 2 Department of Bioinformatics, Biocenter, University of Wuerzburg, Wuerzburg, 97074, Germany; 3 EMBL, Structural and Computational Biology Unit, Heidelberg, 69117, Germany

## Abstract

Software design and sustainable software engineering are essential for the long-term development of bioinformatics software. Typical challenges in an academic environment are short-term contracts, island solutions, pragmatic approaches and loose documentation. Upcoming new challenges are big data, complex data sets, software compatibility and rapid changes in data representation. Our approach to cope with these challenges consists of iterative intertwined cycles of development (“ *Butterfly*” paradigm) for key steps in scientific software engineering. User feedback is valued as well as software planning in a sustainable and interoperable way. Tool usage should be easy and intuitive. A middleware supports a user-friendly Graphical User Interface (GUI) as well as a database/tool development independently. We validated the approach of our own software development and compared the different design paradigms in various software solutions.

## Introduction

Typical challenges in bioinformatics in an academic environment include “ad hoc” programming. No maintenance is really possible as scientists such as PhD students and post-doctoral scholars leave after their thesis is completed or after their post-doc contract. These scientists may also have no formal computer science training, and often there is no structured programming ^1^ and solutions might not be compatible with each other. Furthermore, in an academic environment there are a number of inherent pressures to develop pragmatic and fast (“quick and dirty”) software solutions ^2^.

In addition, there are some new and “modern” challenges, which become more and more pressing simply as the technology progresses: big data ^3^, the wave of “omics” data to process, and the problem of interoperability of software tools ^4^. Well-known recent solutions for this challenge are Taverna ^5^ and Galaxy ^6, 7^. The latter in particular is well suited to dealing with large quantities of data such as new large-scale sequencing data.

Another issue is that the data should be accessible, with uniform syntax and rich semantics for integration. Furthermore, data schemes are prone for change due to rapid advances in the field, so a schema-free representation of data is increasingly important (for scientific data). The UniProt Consortium ^8^ for instance has recently shifted from the use of relational databases to the semantic web for flexible data management.

To counter these older and general as well as new challenges, we have now developed a solution of iterative and intertwined development cycles ( *Butterfly* model), which improves the typical aspects of long-term sustainability and maintenance. Furthermore, it features detailed user-requirement analysis, good graphical and simple user interfaces (optimized human-computer interface, HCI) and intuitive software use that also exploits natural language processing. Our model tackles current challenges: first, the interoperability of the software takes into account middleware solutions, so that both database and user interfaces can be used flexibly. Second, the *Butterfly* approach improves the meticulous database engineering required to build large-scale and “omics” databases.

The *Butterfly* development cycle is different from previous approaches ^9– 12^. It requires some additional time investment at the start for design and implementation, but this pays off later. The *Butterfly* model worked well in our hands regarding the above challenges (section: Real time examples using *Butterfly*). In the following we confirm this by describing concrete software development.

The basic concept is really simple: plan ahead, back-check the critical development steps by a separate sub-cycle and talk with the user. It is important to spend more time on requirement analysis, as well as to invest well in interoperability and maintenance. We do not claim that these problems have not been recognized before nor that no alternative solutions for this are available ^2, 6– 8^, but we are confident that with our approach it will be possible to obtain particularly well optimized and high quality bioinformatics software solutions in an academic environment. The initial time investment in the *Butterfly* paradigm helps to save time later due to the interoperability and easy maintenance of the software solutions achieved.

This paper is organized as follows: this section sets the stage for our agenda (section 1); Current Software Engineering and Development (section 2) highlights the modular phases of software development processes and compares several typical software strategies highlighting the novelty of our approach; next, the *Butterfly* work flow is explained (section 3), and software examples using the *Butterfly* approach (section 4) validate the *Butterfly* design principle by providing concrete examples of software projects from own work. Moreover we discuss some bioinformatics tools based on their type, methodology and usage.

## Current Software Engineering and Development

Software Engineering (SE) ^9^ is one of the most recognized fields in computer science as it matures and expedites the processes of software development. In particular, it allows a focus on the life cycle of software and sustainable development as an improvement to pragmatic short-lived implementations. SE has introduced many process improvement models and techniques ^10– 12^, and Software Development Life Cycle (SDLC) models ^13^, with some variabilities ^14^ and commonalties ^15– 19^. In general, depending upon the observed commonalties, we state

“ *Software Engineering is an integrated, cyclic and product line combination of following independent modular approaches: requirements engineering* ^20– 22^, *design modeling* ^23– 25, 27– 35^ *programming, testing and deployment*”.

**Figure 1.**
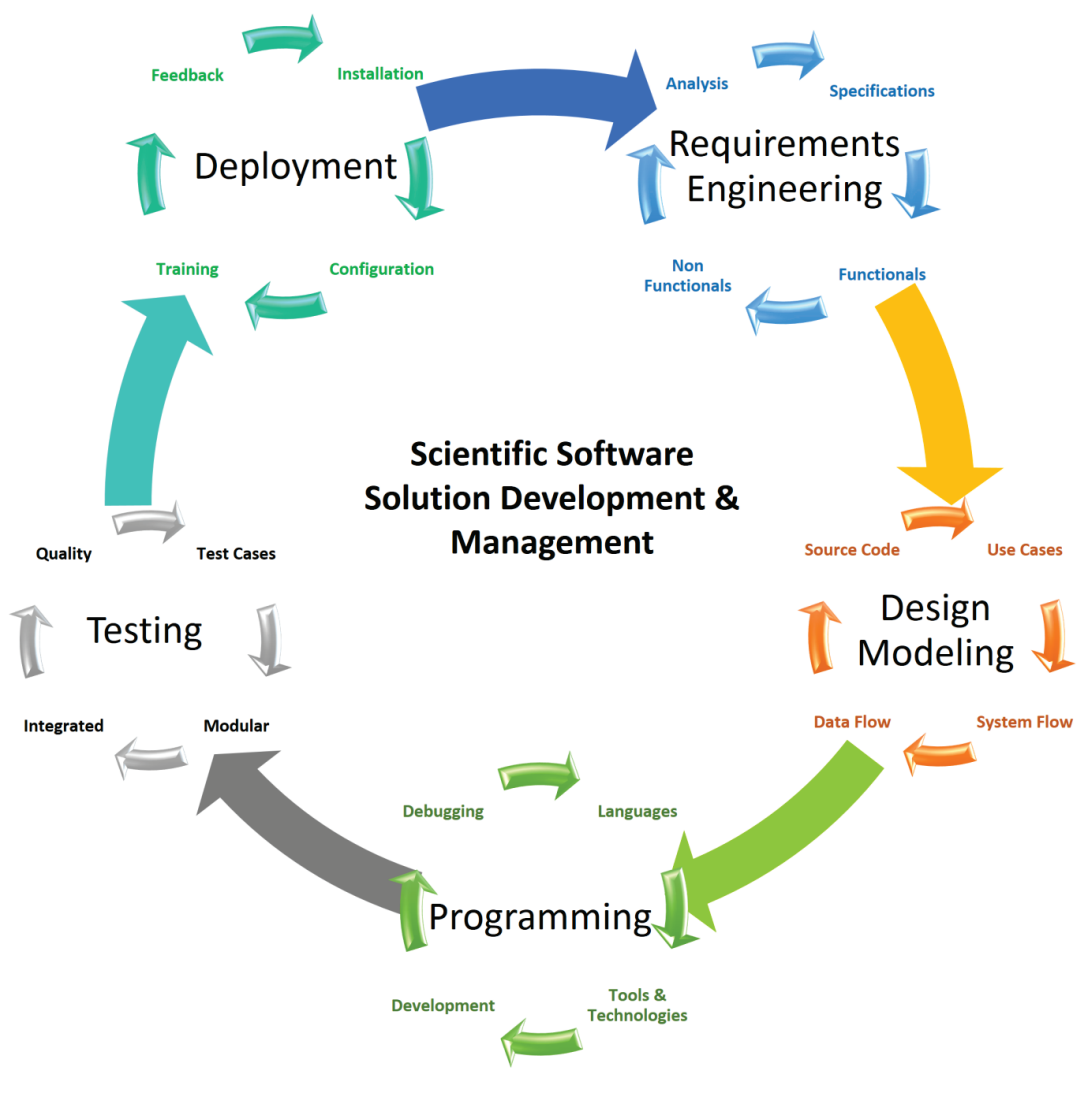
Scientific Software Engineering (SSE). SSE integrates and combines in a development cycle the following independent main modular approaches: requirements engineering, design modeling, programming, testing and deployment. Each approach consists of its own sub-modular, integrated and cyclic combination of internal phases: requirement engineering consists of specification, functionals, non-functionals, and analysis; design modeling consists of use cases, system flows, data flow and source code; programming consists of languages, tools and technologies, development, and debugging; testing consists of test cases, modular, integrated and quality; finally, deployment consists of installation, configuration, training, feedback. Iterative cycles lead to continuous improvement. Achievements translate the goals in good software.

**Figure 2.**
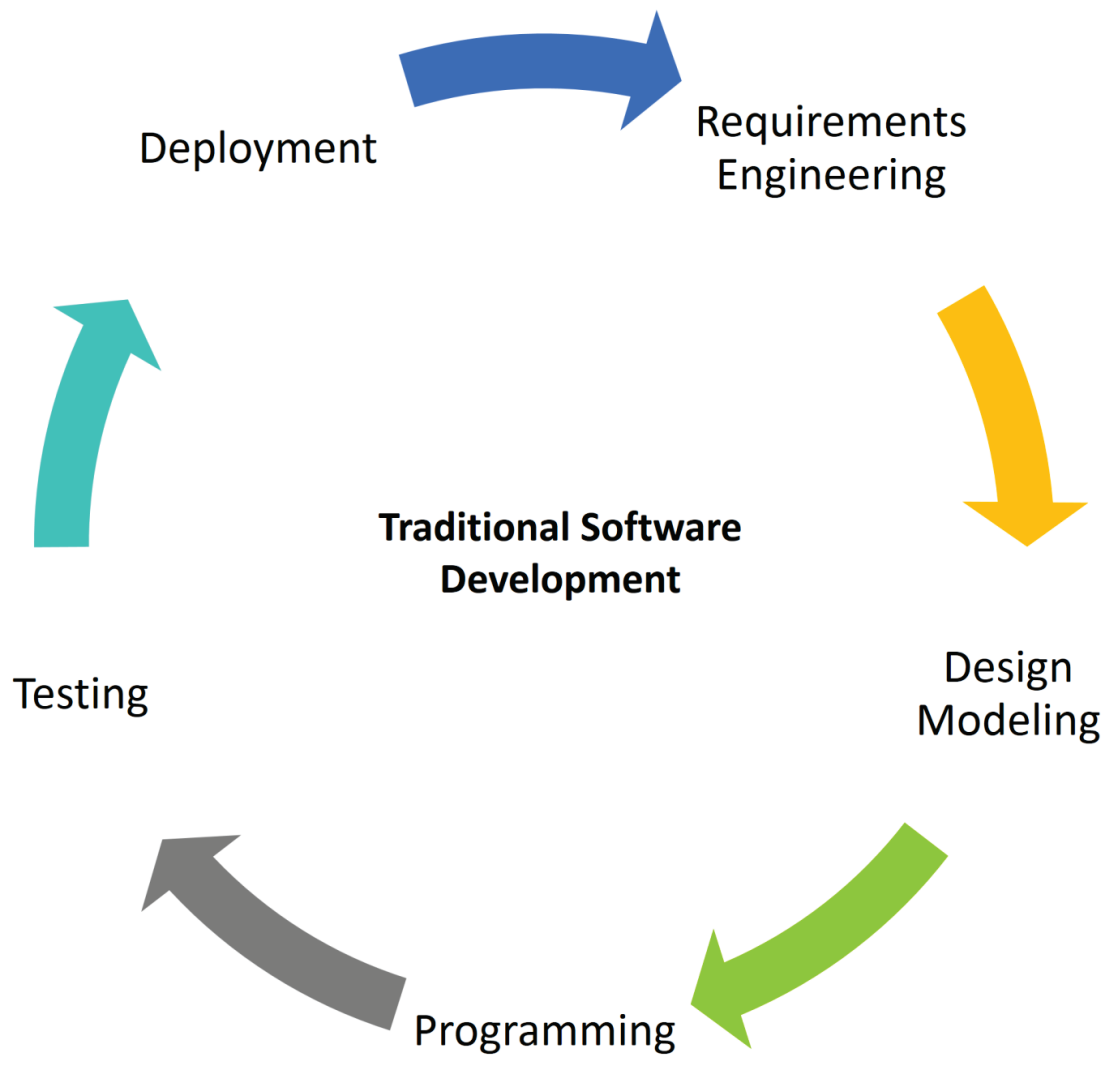
Traditional Software Development consisting of integrated and cyclic combination of the following independent modular approaches: requirements engineering, design modeling, programming, testing and deployment.

The five modular SE approaches remain the same when it comes to the software engineering of the scientific software solution development ( Figure 1). However, in contrast to a pragmatic and maybe traditional software application development in an academic setting ( Figure 2), a major change is the inconsistency in all phases of the SDLC. In the requirement engineering phase ( Figure 2; traditional software solution development), all requirements should be provided before the start of design. This is not the case when dealing with most of the scientific software applications, and the requirements continuously change with the passage of time (we have proposed an updated SSE SDLC Model, Figure 1; scientific software solution development). Ultimately, this complicates the process of analysis and filters out functionals. Programming structures become complex ( Figure 1), as the possibilities of error proneness (both logical and syntax errors) increase due to the continuous increment of variabilities in the pre-processed source code ^15– 19^.

Testing of integrated and individual modules becomes time consuming ( Figure 1), as new test cases have to be continuously rewritten and their application often leads to ‘ripple effects’ ^29^: these are unidentified logical or syntax errors in the system which arise while fixing the errors ^36, 37^. Depending upon the nature of the system, many approaches have been proposed to improve software quality control processes ^38– 50^ which improve standard software development and are important in scientific software quality assurance and improvement. Furthermore if the system keeps changing and is inconsistent, then the deployment procedures can also be complex and time consuming, especially for large applications with multiple interfaces and controls providing numerous individual and integrated functionalities.

To further help (SSE, non-computer scientist Bioinformaticians *etc.*) in expediting the processes of adopting the concepts of SDLC, we provide a tabular comparison between different SDLCs, based on their commonalities and variabilities ( Table 1). This comparison is based on following twenty four defined comparative SDLC (authors’ initiated) measures: *Software Engineering Approach* ^9^, *Initial, Developmental Plan, Software Requirements Engineering, In Depth Requirements Analysis* ^51^, *Requirement Validation, Functionals, Risk Analysis, Software Design, Software Architecture Design, In Depth Software Design Modelling, Reusable Designing, Developmental Plan, Tools and Technology Selection and Analysis, Graphical User Interface Design, Preprocessed Source Code Writing* ^15– 18^, *Integrated Programming, Software Testing, In Depth Software Testing, Customer’s Evaluation, Deployment Procedures, Maintenance, Software Re-Engineering* ^52^, *Cyclic or Repetitive, Easy to learn and Use,* and *User Training*.

**Table 1.** Comparative feature based analysis between different software development life cycle models: *Waterfall Model, V-Model, Spiral Model, Iterative and Incremental Model, Rapid Prototype Model, Extreme Programming Model, Evolutionary Model, Agile Development Model, Code and Fix Model*.

Features/SDLCs	Waterfall	V	Spiral	Extreme Prog.	Iterative	Rapid Prototype	Evolutionary	Agile Dev.	Code & Fix
**Software Engineering** **Approach**	Yes	Yes	Yes	Yes	Yes	Yes	Yes	Yes	Yes
**Initial, Developmental** **Plan**	No	No	No	No	Yes	No	No	Yes	No
**Software** **Requirements** **Engineering**	Yes	Yes	Yes	Yes	Yes	Yes	Yes	Yes	No
**In Depth** **Requirements** **Analysis**	Yes	Yes	Yes	No	No	No	No	No	No
**Requirement** **Validation,** **Functionals**	No	No	Yes	No	No	No	No	No	No
**Risk Analysis**	No	No	Yes	No	No	No	No	No	No
**Software Design**	Yes	Yes	Yes	No	Yes	Yes	Yes	Yes	No
**Software** **Architecture Design**	Yes	Yes	Yes	No	No	No	No	No	No
**In Depth Software** **Design modeling**	Yes	Yes	No	No	No	No	No	No	No
**Reusable Designing**	No	No	Yes	Yes	Yes	No	Yes	No	No
**Developmental Plan**	No	No	Yes	No	No	No	No	No	No
**Tools and** **Technology Selection** **and Analysis**	No	No	No	No	No	No	No	No	No
**Graphical User** **Interface Design**	No	No	No	No	No	No	No	No	No
**Preprocessed Source** **Code Writing**	Yes	Yes	Yes	Yes	Yes	Yes	Yes	Yes	Yes
**Integrated** **Programming**	No	Yes	No	No	No	No	No	Yes	No
**Software Testing**	Yes	Yes	Yes	Yes	Yes	No	Yes	Yes	Yes
**In Depth Software** **Testing**	No	Yes	Yes	No	No	No	No	No	No
**Customer’s** **Evaluation**	No	No	No	No	No	Yes	No	No	Yes
**Deployment** **Procedures**	No	No	No	No	Yes	No	Yes	Yes	No
**Maintenance**	Yes	No	No	No	No	No	No	No	Yes
**Software** **Re-Engineering**	Yes	No	Yes	Yes	Yes	No	Yes	No	No
**Cyclic or Repetitive**	No	No	Yes	Yes	Yes	No	Yes	No	Yes
**Easy to learn and** **Use**	Yes	Yes	No	No	Yes	No	Yes	Yes	Yes
**User Training**	No	No	No	No	No	No	No	Yes	No

We applied these measures to nine different software development life cycle models: *Waterfall Model* ^53^, *V-Model* ^54^, *Spiral Model* ^55^, *Iterative and Incremental Model* ^56^, *Rapid Prototype Model* ^57^, *Agile Development Model* ^58^, *Extreme Programming Model* ^58^, *Evolutionary Model* ^59^, *Code and Fix Model* ^60^. From this comparative analysis we conclude that there is no such one specific SDLC which can be helpful in all required phases of scientific software solution development, but some which might be more useful: *Spiral*, *Waterfall* and *V-Model*. Moreover the SDLCs famous for the quick development ( *Rapid Prototype Model, Agile Development Model, Extreme Programming Model, Evolutionary Model, Code* and *Fix Model*) are very helpful in script writing and fast prototype production; however full sustainability remains nevertheless a challenge.

As this is a general and open comparison, depending on the nature of the scientific software application, one can further analyze and pick that which suits best. Furthermore, we considered only the typical effort and strengths for each of these software development paradigms. A meticulous developer can of course take special care and spend more time on any of the features not typically covered by the software paradigm he follows, and turn the “no” for this feature into a “yes” simply by this additional effort during SDLC (for instance, regarding agile programming – for that matter, extreme programming can also be considered as a type of agile development). The goal for our “ *Butterfly*” paradigm is a SDLC paradigm that fulfils all of the features regarding life cycle management of the resulting software.

## *Butterfly* in scientific academia

If we search for “bioinformatics tools” over the web, thousands of entries can be found at one hit. But how many of those are fully designed, developed and tested solutions, used and maintained for a number of years and still in functional use?

At the beginning of a software project in academia, scientific solution development seems very interesting, fascinating and exciting. But with the passage of time, when the levels of complexities increase (due not only to the lack of developmental skills but also to the unavailability of proper designs), the work starts becoming tedious and unfruitful. This causes a lack of interest in software solution development and leads to a preference for wrapping up the work with a working script or small application, which can be published later on.

**Figure 3.**
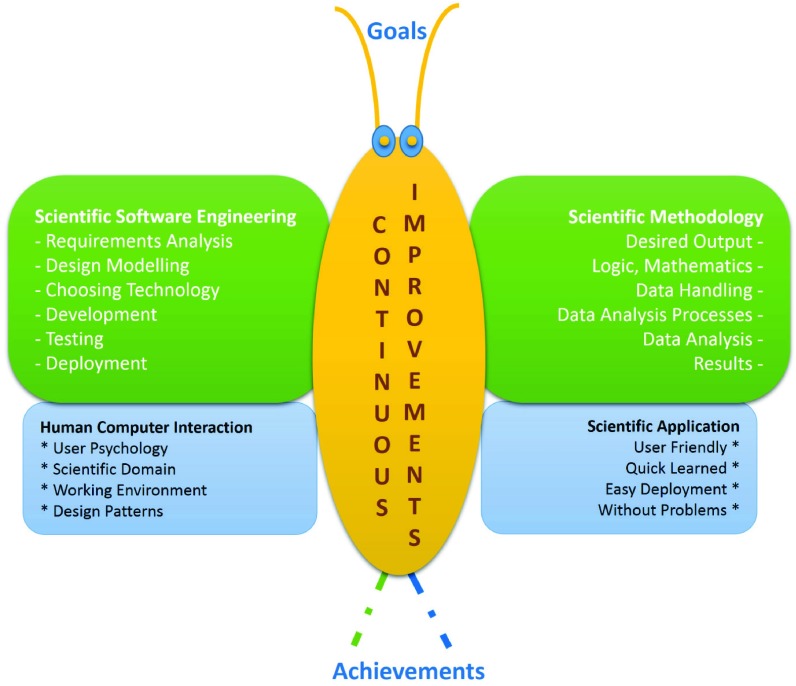
Butterfly model. It consists of four wings: Scientific Software Engineering (upper left), Human Computer Interaction (lower left), Scientific Methodology (upper right) and Scientific Application (lower right). Moreover it leads to continuous improvement (in yellow). The achievements translate the goals into software.

Here we propose a new science-oriented model ( Figure 3), which can help the scientific software solution developers as well as the scientists/end users by generalizing the use of major developmental aspects correlating to the important scientific needs of the target system. The name of our new model is “ *Butterfly*”.

In accordance with the name of the model, the “ *Butterfly”* represents sustainable and continuous improvements from goals to achievement as the central backbone of development. For larger projects this is further improved by using middleware between user interfaces and accessing the various data and databases involved. This also has the advantage that natural language processing needs to be implemented only once in the middleware and all the other modules have access to it. Finally, the backbone arrangement with a powerful middleware as well as all development elsewhere in the “ *Butterfly*” stresses the interoperability of the software. It is developed by using well-defined and compatible output formats. Furthermore, below the middleware, well adapted, interoperable data schemes boost sustainable development of database structures, including efforts for scheme-less databases and other semantic web developments. The four continuously moving wings ( Figure 3) represent the change in the requirements. The upper left wing of the model represents the scientific software engineering principles, and the lower left wing represents the HCI. The upper right wing represents the implementation of the specific methodology (focused on lowering the risk of development ripples) and the lower right wing represents the target scientific application producing the required results.

### Scientific Software Engineering and Scientific Methodology

SSE is the most important phase of the *Butterfly* model, which promotes the usage of any earlier mentioned SDLC involving requirements analysis, design modeling, programming and testing of the scientific software solution. It correlates with the phase Scientific Methodology; the finalized functional requirements are based on the desired system output, and the system should be modeled according to the defined logics and mathematics (individual as well as the sequence of algorithms if there are more than one). The most suitable, advanced, recent, economically affordable, transferable, flexible and reliable developmental technologies should be chosen considering the use and availability of the data (large, small, complex, shared *via* intranet or internet).

Meanwhile, programming and processing of the complex and large data should be undertaken in order to have efficient data analysis, management and visualization. During the testing procedure of the developed system, all modules should be properly tested by the developer, by testing experts - if available -, and by the appropriate users. While testing the newly developed scientific system, the system should not be considered ready or functioning straight away. No real experiments should be performed prior to through testing to avoid any loss of data or waste of any scientific research/biological material or living beings (especially in case of behavioral research and analysis). Only after successful deployment the real time results should be evaluated.

For instance, if the target scientific software solution is a database manipulation and management system, then it will require to properly model the database schema (entity relationship model), by reducing the levels of data redundancy and dependency, *via* data normalization. There are five data normalization forms: 1NF, 2NF, 3NF, 4NF and 5NF, which include conceptual procedures for comprehensive database designing ^61^. These data normalizations help in shaping the data types (1NF), developing the relationships between non-key and key fields (2NF, 3NF) ^62, 63^, and dealing with multi-valued facts which correspond to many relationships (4NF and 5NF) ^64, 65^. Structured data are more helpful in case of search and indexing operations than simple databases with entities but without hierarchal relationships. Moreover, if the experimental data are well normalized, then in case of large datasets, they will expedite the processing speed and reduce time in searching and analyzing the elements.

### Human computer interaction and scientific applications

The lower left wing of the *Butterfly* ( Figure 3) is the HCI, known as Human Machine Interaction (HMI) ^66– 68^. This interacts with the lower right wing *i.e.* Scientific Application. HCI defines the implementation of the mechanisms that establish the efficient communication protocols between human and machines. These protocols are based on the textual, visual, sensory, video, audio and event based information, provided by both the user and the machine (computer). The backbone of the *Butterfly* allows by its middleware to rapidly exchange GUI applications and accessed databases if there is a need for it, considering the rapid developments in bioinformatics or if a user wishes to use GUIs or databases from comprehensive software environments, for instance regarding large-scale sequencing from Taverna ^5^ or Galaxy ^6, 7^.

Unfortunately HCI was the most ignored and unattended phase of scientific software solution development. This starts not to represent the state of the art any more as the awareness on HCI is increasing ^85^, for instance EBI has specialized UX personnel who can be engaged in projects. Nevertheless, often developers do not give priorities to the GUI design and implementation. The reasons for this negligence could be the pressure due to time limits for development, rapid functionality addition during development, excessive iterations, less field knowledge, lack of awareness about the importance of HCI, competitive general purpose software and human behavior analysis *etc*. ^66^. In principle and practice, with respect to the user’s point of view, HCI is one of the most important parts of the software development. If the HCI is bad and the software is not easy to use, why use the software at all? Below we will present a particularly well-engineered solution in this respect, the ant database, focusing on a really easy user database access by smart phone.

During the scientific solution deployment and usage, the end user is probably a scientist without strong informatics background. To run and execute a script, first the compiler and the editor need to be installed, then depending upon the needed external libraries, the necessary libraries will need to be identified and used. This might be a very hectic task, especially for a person with no informatics background. In most of the cases, the result-producing applications cannot be used because of difficulties in deployment and execution procedures. Moreover, most of the time, many scientific software applications are not well documented, which also increases the level of complexities at the user end.

If the application is configured and is executed successfully, then the next task is to use it. If the application has been developed with an unfriendly GUI or a command line interface, it might be a problem for the user scientist, as to how to use it. If the application is not easily deployable and useable, then in most of the cases it can only lead to the loss of developer’s efforts.

To avoid such problems, the following aspects should be considered: user psychology, scientific domain, working environment and HCI design patterns ( *Window Per Task, Interaction Style, Explorable Interface, Conversational Text, Selection, Form, Direct Manipulation, Limited Selection Size, Ephemeral Feedback, Disabled Irrelevant Things, Supplementary Windows and Step-by-Step Instructions*). It is very important to understand the common psychology of the users of the applications *e.g.* if they are the laboratory scientists, they will be happy to have user friendly graphical interface and easy deployment procedure ( *e.g*. small setup which runs and automatically configures all the required settings in the user’s system), so that they do not have to spend additional time on the configuration.

Scientific domain and working environments are two more important aspects to be considered while designing the graphical interface. These are particularly important especially when developing real time systems *e.g.* embedded, robotic, mobile applications. Moreover, it is important to consider that the use of HCI patterns and principles (Cooperation, Experimentation, Contextualization and Iteration) have a reliable HCI communication protocol implementation.

In general, non-functional requirements specify the overall objectives of the system together with the information about quality attributes ( *e.g.* *Performance, Operating, Platform, Modifiability, Portability, Reliability, Security, Usability* *etc.*), and functional requirements explain the sets of input, output and behavior of the system. Here, we are somewhat extending these general concepts: the functional requirements can also be those which can be implemented (based on existing resources, time, budget, labor, tools, technologies and methodologies), and non-functional requirements can also be those which cannot be implemented but which are qualitatively helpful in defining the system and its usability. It is very important to iteratively clarify with the users what will be the expected end-product because it is possible that the user may not like the output of the system after development cycles. If the end product is unsatisfactory in this respect, all the efforts will have been in vain.

After designing an interface, one of the important tasks is to evaluate its effectiveness and potential. One general and effective way is to engage the users and consider their feedback at every step. The technical approach is to use the HCI design principles: Experimentation, Contextualization, Iteration and Empirical Measurement ^67^. Another beneficial method is to adopt and use the HCI design patterns: Window Per Task, Direct Manipulation, Conversational Text, Selection, Form, Limited Selection Size, Ephemeral Feedback, Disabled Irrelevant Things, Supplementary Window and Step-by-Step Instructions ^67^.

## *Butterfly* workflow designs

**Figure 4.**
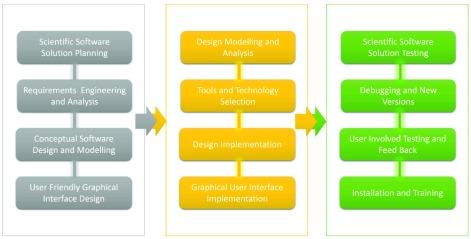
Butterfly three layer model. Shown in gray is the abstract layer, in yellow is the basis for design and development, and in green the implementation and testing by the user. The software is released including installation and training.

The *Butterfly* model implementation mechanism has a three-layered architecture: Gray, Yellow and Green ( Figure 4).

### Gray layer

The gray layer represents the most important phase of scientific software solution development, which involves software designers, developers, testers, graphical interface designers and, most importantly, the users. It consists of four phases: scientific software solution planning, requirements engineering and analysis, conceptual software design and modeling, and user friendly graphical interface. The layer has been named ‘Gray’ because at the beginning of a new scientific software solution development, most of the information seems uncertain.

**Figure 5.**
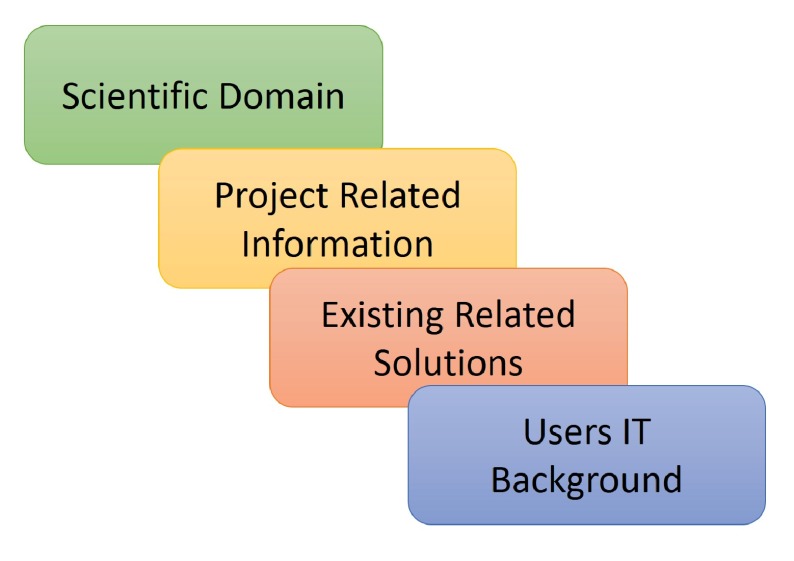
Scientific software solution planning. Abstract planning is the first step of the top, abstract layer (gray, Figure 4) of the Butterfly design, key steps are indicated.

**Figure 6.**
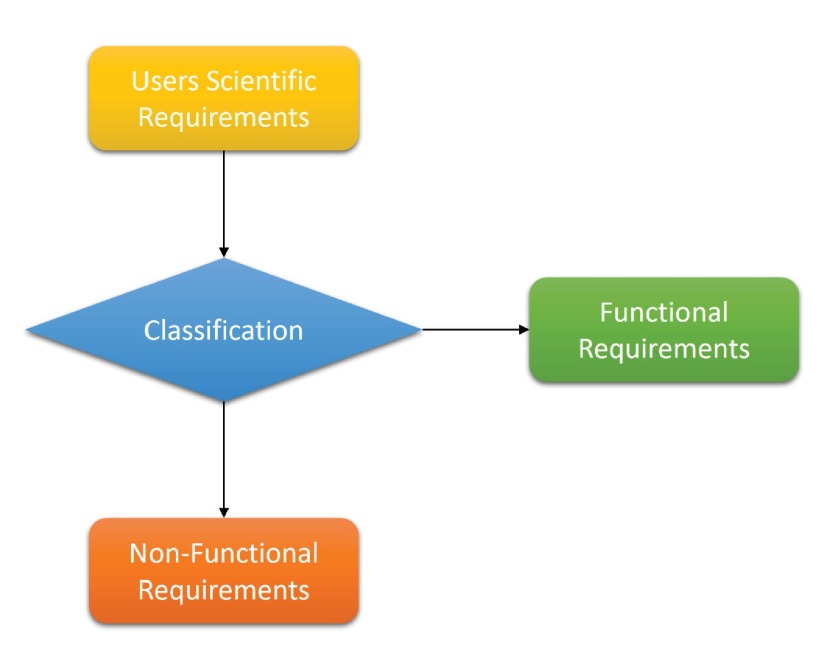
Requirements engineering and analysis. This is the 2 ^nd^ step of the top layer (gray layer) of the three layer model in the Butterfly design. Key tasks are indicated.

Scientific software solution planning ( Figure 5) is the first step towards a new scientific application development, which requires the introduction to the field itself ( *e.g.* biochemistry, neurobiology, genetics, metabolomics, proteomics *etc.*) and project related information ( *e.g.* what could be the end product, input to the system, expected output from the system, methodology, ideas, opinions *etc.*). It is important to know about the user’s information IT background and existing already available (old and recently developed) scientific solutions to the problem. The next important phase is to perform requirements engineering and analysis ( Figure 6). During this phase, the most important tasks are to gather the requirements from users ( *e.g.* interviews, brainstorming, documents, publications, running related systems based information *etc.*) and classify the information in functional and non-functional categories. Here, function requirements are those which can be implemented (based on existing resources, time, budget, labor, tools, technologies and methodologies), and non-functional requirements are those which cannot be implemented. It is very important to clarify with the users what will be the expected end-product because it is possible that the user may not like the output of the system after development. In this case, all the efforts will have been in vain.

**Figure 7.**
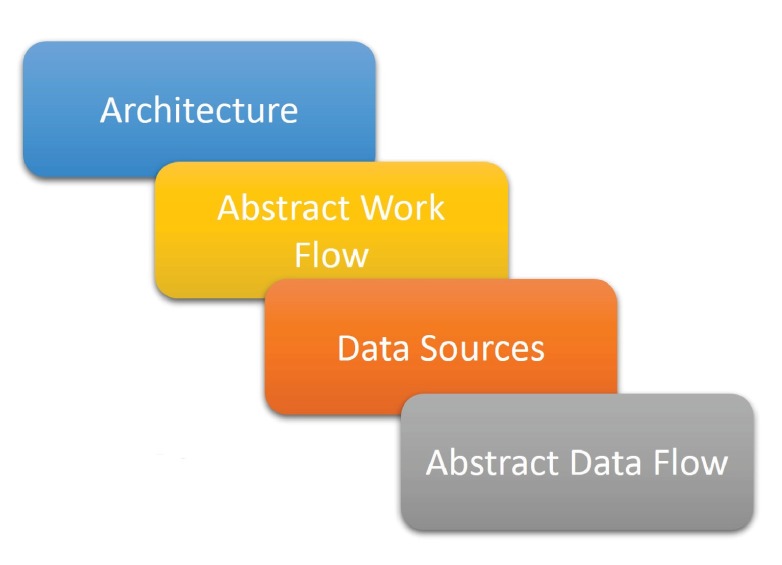
Conceptual software design and modeling. This is the 3 ^rd^ step of the top layer in the butterfly model.

The third phase is the conceptual software design and modeling ( Figure 7). This is particularly important when there is a team of software developers. Before moving ahead, one should go for some abstract designs based on functional requirements and discuss these with the team. It is crucial to estimate the expected workflow, data sources and data flow in the system. If possible, the abstract design should be discussed with the users as well.

**Figure 8.**
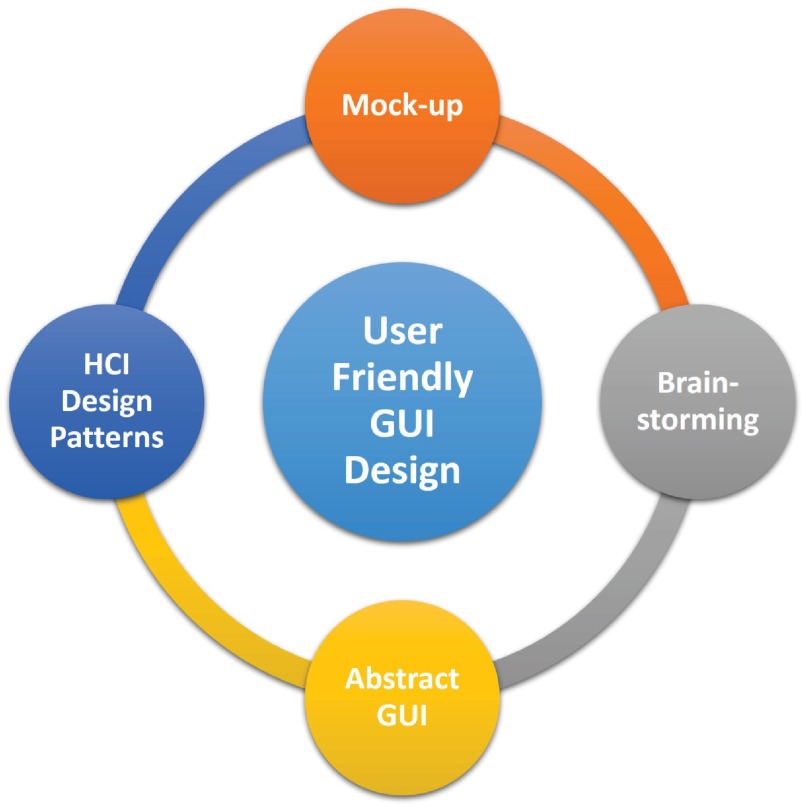
User Friendly Graphical Interface Designing. This is the final step of the top, abstract layer (gray) of the three layers in the butterfly design.

The last phase of the gray layer concerns the design of a user-friendly GUI ( Figure 8). First, some mock-ups should be made (hand-made on papers or better to use white board with color markers and later make pictures of finalized GUI designs), then these should be discussed with users in a brain storming session. Finally, based on the perceived designs, the abstract GUI (GUI with no running functionalities) should be created using HCI design patterns.

***Case study***. We have successfully applied the Butterfly model in the implementation of newly proposed applications i.e. DroLIGHT ^71– 73^, scientific computational solution towards neurobiology and photobiology ( Figure 10). The engineering processes started with the initial scientific software solution planning, the involvement of the members of a scientific group (Department of Neurobiology and Genetics, Biocenter, University of Wuerzburg, Germany). The desired end product was a system towards the behavioral biology of the fruit fly. The overall requirements were about to implement a domain specific, intelligent, distributed, real time, embedded, data management system capable of controlling hardware devices, proficient in producing different colors of lights and monitoring the movements of *Drosophila melanogaster*. Moreover, it should be capable of generating circadian and diurnal rhythms, experimental data management system and visualize experimentation’s output in two and three dimensional graphics formats.

We analyzed and distinguished between functional and non-functional requirements to draw the conceptual models of the proposed system. Furthermore, we involved the scientific team members in different brain storming session and drawn mockups with the implementation of following design patterns: window per task, direct manipulation, conversational text, ephemeral feedback and step-by-step instructions. The designed mockup was providing multiple instances, allowing users to directly interact with the system using provided controls, offering the textual command line instruction mode for human machine interactions, status updates and tools tips ^71– 73^.

### Yellow layer

The yellow layer involves designers and developers. It consists of four phases: design, modeling and analysis, tools and technology selection, design implementation, and GUI implementation.

During design modeling and analysis, the important task is to create different implantable designs *e.g.* UML models (use case, class, system sequence, activity, component *etc*.) and database schemas (in case it’s a database management system). Here, we strongly recommend the use of Product Line architecture design modeling, where the whole software is divided into sets of modules, which work individually as well as together. This will customize project development and reduce error proneness during development. Moreover it will increase the concepts of modular reusability.

The next step involves the choice of available tools, technologies and programming languages that will be implemented in the designed models. The last step focuses on adding functionalities to the designed GUI.

***Case study***. Meeting the scientific objectives of understanding the light-evoked behaviors of *D. melanogaster*, especially the synchronization of its endogenous clock to light-dark cycles and following the implemented conceptual design and mockups (Gray layer), we constructed the implementable designs of DroLIGHT ^71– 73^. We implemented UML principles and notations for Meta model software designs with abstraction and modification techniques. The designed UML diagrams of DroLIGHT ^71– 73^ described the application’s functionalities, user access, internal work flow, system sequence, pre-processed source code structure, compilation, execution and integration with involved other components ^71– 73^.

We invested time in searching the recent available and reliable technologies, which could be used for the design and development of newly proposed system. We used Astah modeling tool to construct the different UML diagrams and focusing on the functional requirements, we developed DroLIGHT ^71– 73^ (front end and back end) in managed code using C# (object oriented programming language), within Microsoft Visual Studio (Dot Net 2012).

### Green layer

The green layer describes the final *in house* testing and debugging by the developers and tester. Some scientific software applications are developed to process the raw data using mathematical algorithms ( *e.g.* processing GC-MS, LC-MS, NMR data), whereas some applications are implemented to perform different kind of experiments, which in return produce experimental data *e.g*. towards behavioral research on animals and insects *etc*.

Processing raw data is safe even if there are still some problems ( *e.g*. minor calculation mistakes due to the different levels of fractional values or wrong implementation of mathematical algorithms, or some software developmental issues, which could be ‘ripple effects’, or some logical bugs) after testing. However, when applied in real time experimentation, if the software does not work as expected, it could be very expensive and dangerous ( *e.g.* if there is a software to control the temperature and light during experiments on insects or animals, and there are problems during experimentation leading to changes of the normal or expected temperature and light to some extreme positive or negative values, then this could not only effect the system’s hardware but can also threaten the life of animals or insects).

In order to avoid such problems, we enforce test trails by different users before making the software available for installation and training in the public domains.

***Case study***. The implemented version of DroLIGHT (sections: Gray layer and Yellow layer) was tested in the development lab and applied Black box testing, White box testing, Unit testing, Integration testing, Functional testing, System testing, End-to-end testing, Load testing, Stress testing, Performance testing, Usability testing, Install/uninstall testing, Compatibility testing and Comparison testing strategies. Based on the observed problems (errors, bugs, ripples *etc.*) we produced different versions, and kept on testing until a reliable version was achieved. As in agile development, refactoring (improving code quality without changing its semantic meaning) is seen as an effort that copes with ripples and accompanies development. DroLIGHT improvement was achieved efficiently here using windows XP. Using again the Butterfly paradigm, similar results should be obtainable focusing on scrum techniques and iterative lean development with sprint backlogs, but this was not tested by us. Later after training the scientists about to how to use and configure the application, reliable release was shared and tested in the scientific labs.

Constant improvement is further boosted by sustainable data structures and engineered robustness of the application. Furthermore, sustainable development and interoperability are considered from the start, support the rapid further development/improvement of software to study and manipulate circadian rhythm by different light cycles and test environments in *D. melanogaster*.

## Real Life Examples using *Butterfly*

A number of new scientific software applications based on the concepts of the *Butterfly* model have already been proposed, designed, implemented, tested and are currently in use. Some of them have been published and some remain unpublished. These applications are: Least Square MIDA (LS-MIDA), DroLIGHT, Isotopo, Lipid-Pro and App Ant Database.

**Figure 9.**
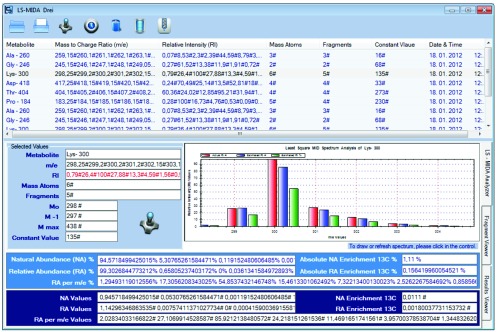
Least Square Mass Isotopomers Distribution Analysis’s (LS-MIDA) main graphical user interface. Scientific software solution towards bioinformatics and biochemistry which estimates mass isotopomers distribution from spectral data by analyzing each peak of given mass and each mass atom fragment. ( http://www.tr34.uni-wuerzburg.de/computations/ls_mida/).

LS-MIDA ^69, 70^ ( Figure 9) is an own published scientific software (Department of Bioinformatics, Biocenter, University of Wuerzburg Germany) which estimates mass isotopomer distribution from the spectral data by analyzing each peak of given mass and each mass atom fragment. It implements a chain of mathematical and statistical algorithms, provides graphical interfaces to help users analyze experimental data and visualize the results, and provides a third party independent experimental data management system. For the most parts the requirement engineering and software design steps are followed until the final solution presented ( Figure 9), and details on tested earlier solutions or specific requirements are available from the authors.

**Figure 10.**
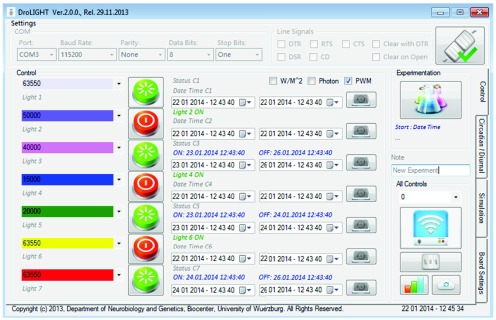
DroLIGHT’s main graphical user interface. It is a scientific software solution towards neurobiology and photobiology, capable of controlling and automating the hardware that produces different colors of lights *via* Light Emitting Diodes (LEDs). It provides experimental data management system, circadian and diurnal rhythm generation, 3D visualization of system’s performance and experimentation details ( http://www.neurogenetics.biozentrum.uni-wuerzburg.de/en/project/services/drolight/).

DroLIGHT ^71– 73^ ( Figure 10) is an earlier described (section: *Butterfly* workflow designs) scientific solution to control the irradiance and wavelength of light designed for the photo-receptor system of the fruit fly.

**Figure 11.**
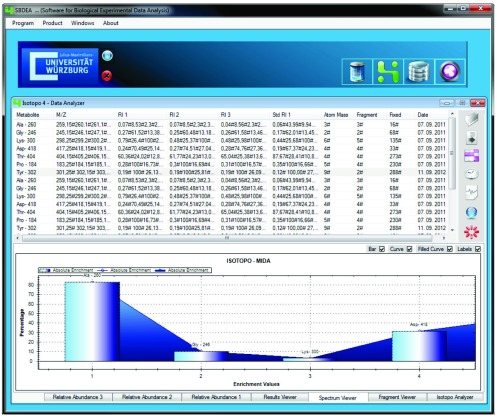
Isotopo Data Analyzer’s main graphical user interface. Scientific software solution towards bioinformatics and biochemistry, with the ability of performing quantitative mass spectrometry to mixtures of materials labeled with stable isotopes. It provides internal database management system, third party independent file based experimental data management system and intelligent data format parser for data extraction and conversion of different data formats. ( http://spp1316.uni-wuerzburg.de/bioinformatics/isotopo/).

Isotopo software ^74, 83^ ( Figure 11) is a published scientific software (Department of Bioinformatics, Biocenter, University of Wuerzburg, Germany), a bioinformatics solution with the ability of performing quantitative mass spectrometry in isotope labeling experiments. It is an extended version of the earlier software LS-MIDA with a well-optimized mathematics implementation. It not only provides the graphical interfaces for gas chromatography-mass spectrometry (GC-MS) experimental data analysis, visualization and management, but also provides an intelligent data parser to automatically transform the machine’s pre-processed data into the processable format of the Isotopo software (reducing both time and labor). It also provides a complete database management system as a simple, well sustainable version of a middleware between data storage and GUIs.

**Figure 12.**
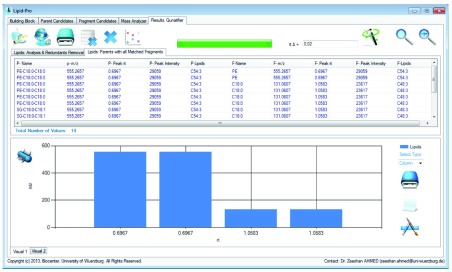
Lipid-Pro’s main graphical user interface. Scientific software solution towards lipidomics and pharmaceutical biology ( http://www.neurogenetics.biozentrum.uni-wuerzburg.de/en/project/services/lipidpro/).

Lipid-Pro ( Figure 12) is a scientific software (computational) solution towards lipidomics and pharmaceutical biology (Department of Pharmaceutical Biology Department of Neurobiology and Genetics and Department of Bioinformatics, Biocenter, University of Wuerzburg, Germany). It is a new solution towards the lipidome analysis including retention time (RT), mass to charge ratio values (m/z) of precursor and fragment ions, chemical compositions and peak intensities. Moreover, it provides comprehensive spectral data management, sharing and integration features.

**Figure 13.**
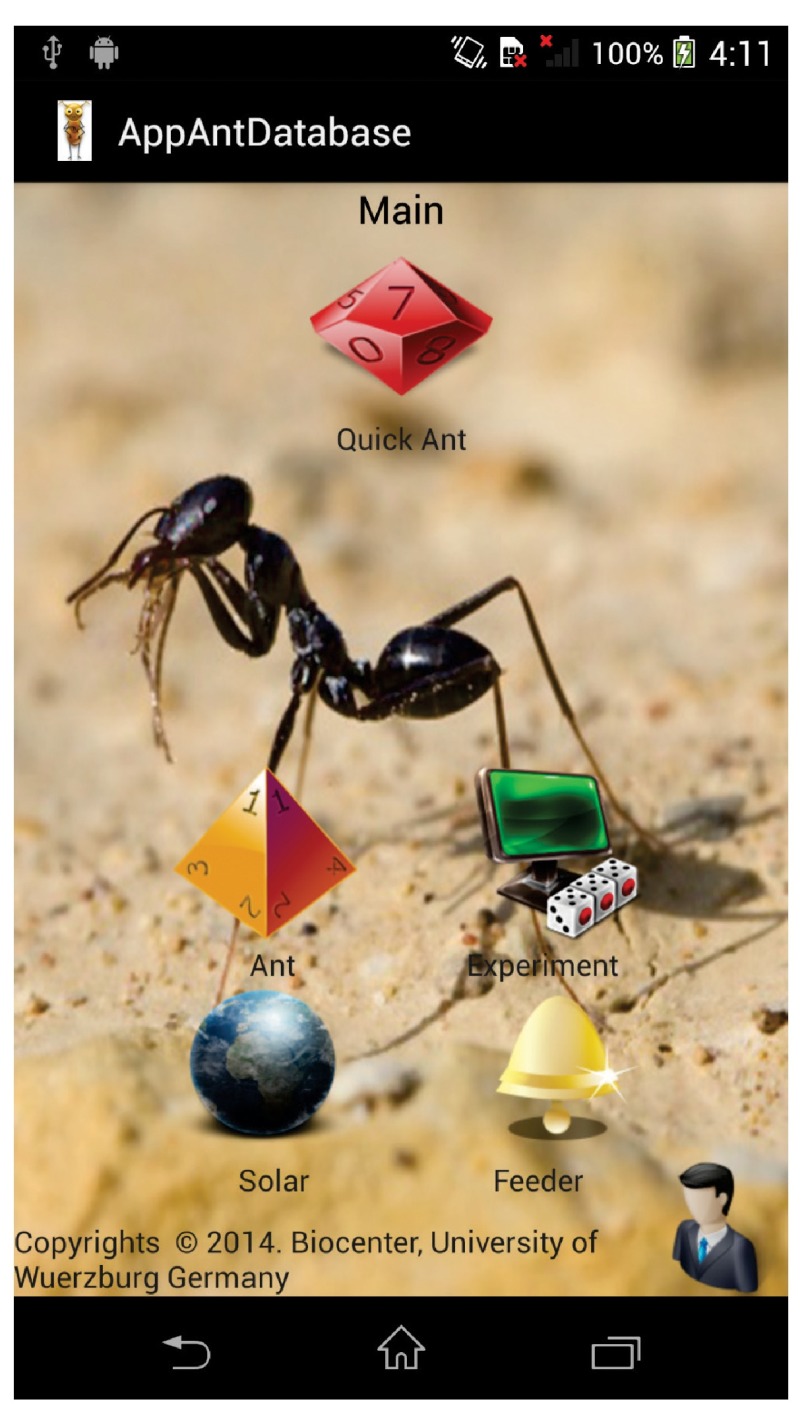
App Ant Database’s smart phone graphical user interface. Scientific software solution towards the experiment data management during experimentation on desert ants. It offers user friendly graphical interfaces for the experimental data entrance, manipulation, management and sharing ( http://www.neurogenetics.biozentrum.uni-wuerzburg.de/en/project/services/ant_app_db/).

App Ant Database ^84^ ( Figure 13) is a scientific software solution, featuring a distributed and embedded database system in the form of a smart phone, tablet and desktop application towards experimental data management and approximate solar estimations during experimentation on different insects (Department of Behavioral Physiology and Sociobiology, Biocenter, University of Wuerzburg, Germany). It is unique and the first bioinformatics smart phone application to be used in the deserts for the behavioral experiments. After extensive requirement engineering, we established an extremely easy to use graphical interface. Furthermore, after studying the user requirements in monitoring desert ant movement and orientation in the desert, the application not only automatically records ant movements, but also estimates and calculates automatically all additional variables required for the project such as azimuth, solar time, equation of the time, time offset, hour angle, altitude, sunrise, sunset and solar noon using astronomical algorithms, recommended by the National Oceanic and Atmospheric Administration (NOAA).

Regardless of the individual specifications, development details, technologies used and usage perspectives, the key principles of the *Butterfly* model were applied in the design of these software solutions. Although the requirements for each of these were not fixed in the beginning, comprehensive requirements gathering and analysis operations were performed using brain storming and interviewing methods. Based on filtered out the functional requirements, the most suitable SDLCs (V-Model ^54^ and Spiral ^55^) were applied and the software applications were designed using UML (including use cases, class diagrams, sequence diagrams, work flows, activity flows, components diagrams, data flow diagrams *etc.*). Of those SDLC’s used in scientific software solution development, the Spiral Model proved most helpful and best suited due to its four main pillars: determine objectives, identify and resolve risk, development and test, and plan the next iteration. Another advantage of the spiral model is its risk driven approach, incorporating many useful features and refinements of other software development life cycle models ^54, 72^.

Using the HCI design patterns and principles, the graphical user interfaces of all these applications were designed considering the psychology, scientific and informatics backgrounds of the end users and the deployment environments.

All these applications are easy to deploy and use. We found that users did not require training to install, run and use the applications. As scientific research is a never ending process, these applications are still in development and will be continuously improved with respect to the methods, features, performance and technologies.

## Comparing bioinformatics tools

We have performed a short comparative analysis of some bioinformatics software applications (C13 ^75^, Metatool ^76^, BioOpt ^77^, FiatFlux ^78^, ReMatch ^79^, Biolayout ^80^, LS-MIDA ^69, 70^, DroLIGHT ^71– 73^, Isotopo ^74, 83^), describing their type, methodology, implementation, user friendliness, configuration *etc.*, based on the provided, published information ( Table 2).

**Table 2.** Comparative analysis of different scientific software applications. SSE=Scientific Software Application; App.=Application; DB=Database; DM=Data Management; Sys.=System; SDLC=Software Development Life Cycle.

Applications/ Comparative Measures	C13	Metatool	BioOpt	Fiatlux	ReMatch	Biolayout	LS-MIDA	Dro-LIGHT	Isotopo
**SSE?**	Yes	Yes	Yes	Yes	Yes	Yes	Yes	Yes	Yes
**App. Type**	Desktop	Desktop	Desktop	Desktop	Web	Desktop	Desktop	Desktop	Desktop
**Data** **Management**	No DM Sys.	No DM Sys.	No DM Sys.	No DM Sys.	DB	No DM Sys.	File based	File based	File based and DB
**Script or** **Prototype**	Script	Script	Prototype	Script	Prototype	Prototype	Prototype	Prototype	Prototype
**Algorithm** **Type**	Parallel	Sequential	Sequential	Parallel	Sequential	Parallel	Sequential	Parallel	Sequential
**Algorithm/** **Methodology**	Isotopic Labelling	Schuster Algorithm	Mass Balance Equation	Isotopic Labelling	Carbon Mapping	Markov Clustering	Least Square	Circadian Rhythms	Partial Least Square
**Running** **Mode**	Interactive	Interactive	Batch	Interactive	Interactive	Interactive	Interactive	Interactive	Interactive
**Publishing,** **licensing**	Published, Free	Published, Free	Published, Free	Published, Free	Published, Free	Published, Free	Published, Free	Published, Free	Published, Free
**SDLC** **Information**	Not Provided	Not Provided	Not Provided	Not Provided	Not Provided	Not Provided	V-Model	Spiral	V-Model
**HCI** **Information**	Not Provided	Not Provided	Not Provided	Not Provided	Not Provided	Not Provided	HCI Patterns Implemented	HCI Patterns Implemented	HCI Patterns Implemented
**User Friendly**	No	No	No	No	Yes	Yes	Yes	Yes	Yes
**Easy to** **configure**	No	No	No	No	Yes	Yes	Yes	Yes	Yes
**Easy to train**	No	No	No	No	No	No	Yes	Yes	Yes
**Software** **Re-Engineering**	Yes	No	Yes	Yes	Yes	No	Yes	No	No
**Cyclic or** **Repetitive**	No	No	Yes	Yes	Yes	No	Yes	No	Yes
**Easy to learn** **and Use**	Yes	Yes	No	No	Yes	No	Yes	Yes	Yes
**User Training**	No	No	No	No	No	No	No	Yes	No

For our comparison, we chose software applications from fields we are familiar with, yet tried to cover a broad range of different applications and compared Metabolic Flux Analysis (MFA) as well as software metabolic network analysis ^81, 82^, Mass Isotopomers Distribution Analysis (MIDA) including GC-MS data analysis ^69, 70^, and neurobiology applications for behavioral analysis of insects such as desert ants and fly ^71– 73^.

We used the following parameters to classify the chosen bioinformatics software application: SSE type, data management, script or prototype, algorithm type, algorithm/methodology, running mode, publishing, licensing, SDLC information, HCI information, user friendly, easy to configure, easy to train, software re-engineering, cyclic or repetitive, easy to learn and use user training.

From the observed results ( Table 2) we conclude that almost all of the applications have good implementations of their methodology (algorithms *etc*.) but often lack in usage point of views (user interface, documentation *etc*.), or long term sustainable development of the software (at least regarding the organization of future further developments). These shortcomings are prevented when following the “ *Butterfly*” paradigm.

## Conclusions

In the earlier sections of this paper we presented the concepts of usage of the existing scientific software solution design, modeling, implementation, testing and deployment. This helps in resolving conflicts and highlighting valuable differences between the traditional (professional) and scientific applications. This paper also proposes a new approach towards user friendly scientific software solution development, with emphases on the use of proper SDLC, HCI and technologies. The successful implementation of the five applications discussed strongly validates the potential of the *Butterfly* model. We have tried to present a strong case for our *Butterfly* model but it is of course only our own approach to achieve sustainable software development in an academic environment. We clearly see from our development experience that the efforts in preparation and implementing scientific software engineering for an application pay off particularly well if there is a series of programs developed. However, we claim also that one would think more often about such a series of successive improved programs if there is the option to easily implement such an effort, and again the *Butterfly* model would help here.

In conclusion, although the adaptation of SSE principles to the *Butterfly* model may seem to increase developmental work load in comparison to the current running programming method applications, the *Butterfly* model will ultimately reduce the work by making the scientific application well designed, flexible, structured, reusable, developed according to a product line, as well as analytical and of high quality. According to its design, the software developed using the *Butterfly* paradigm is user friendly, easy to learn and deploy.
