# Supplementary material for: Developing sustainable software solutions for bioinformatics by the “ Butterfly” paradigm
Source: F1000Res. 2014 Aug 1;3:71. Originally published 2014 Mar 13. [Version 2] doi: 10.12688/f1000research.3681.2 (PMC4215756; doi:10.12688/f1000research.3681.2)
Supplement: Peer review history

# Peer review: Referee response for version 2

**DOI:** 10.5256/f1000research.5210.r6557

**Published:** 2014-10-28

**Authors:** Wolfgang Mueller

**Affiliations:** 1 Heidelberg Institute of Theoretical Studies gGmbH, Heidelberg, Germany

I have read this submission. I believe that I have an appropriate level of expertise to confirm that it is of an acceptable scientific standard.

# Peer review: Referee response for version 2

**DOI:** 10.5256/f1000research.5210.r5923

**Published:** 2014-08-26

**Authors:** Paul Vauterin

**Affiliations:** 1 Wellcome Trust Centre for Human Genetics, University of Oxford, Oxford, UK

I have read this submission. I believe that I have an appropriate level of expertise to confirm that it is of an acceptable scientific standard.

# Peer review: Referee response for version 1

**DOI:** 10.5256/f1000research.3945.r5147

**Published:** 2014-07-09

**Authors:** Wolfgang Mueller

**Affiliations:** 1 Heidelberg Institute of Theoretical Studies gGmbH, Heidelberg, Germany

Caveat: I lead a group that does scientific software development. I am interested in software development both from the conceptual and the technical side. However, I would not be a serious contender for a software engineering chair. As a consequence, I cannot claim full knowledge about the state of the art of software engineering processes.

I agree to the following takeaways that I pull out of the paper:

- If you know you are building an application which is for other people than yourself, it makes sense to follow a software engineering process.
- Think about maintainability and sustainability of your software.
- Think about your users and write usable software.
- Operate using cyclic refinement

The *Butterfly *model is an assembly of current software practices, emphasizing the needs of the scientific software developer. I see the strength of the article in giving a concise overview of what a scientific software developer should do.

However, I disagree with some statements made in the paper. Some of these statements relate rather to anecdotal experience. This points to one of the weaknesses in the paper: There are statements made with respect to the scientific software domain that are not marked as anecdotal, but which are not backed by either empirical facts (e.g. questionnaires) or pointers to such facts in the literature.

In more detail:

Generally I agree, science is a fast-paced environment with rapidly changing requirements asking for suitable software engineering processes.

*Current software engineering and development*

The paper comprises a large number of citations. However, I do not agree with the insights taken by the authors from that literature.

To my knowledge, the field of Software Engineering has realized that one key factors for success is if software matches the needs of users. Agile Development follows the view that often-times, users don't even know what they need until they are closely involved in development and see where their own ideas of their work (i.e. using the program) could fail. This is why there is less of an initial requirements analysis, but there is a series of sprints that each seek the implementation of features. Each feature implemented also influences the next stage of the requirements analysis.

The authors of this paper see the lack of quality software production as one of the weaknesses of agile software engineering practices. However, Agile software engineering practices like XP and Scrum emphasize the importance of testing and other practices intended to increase the software quality. Such processes definitely do not disregard the production of quality production. So, to cut a long story short, I do not agree with the impression created in the paper that agile processes lead to muddle-through software that is unstable and short term due to the process.

You criticize the "ripples" through the program that come from changes. Ripples is the word describing the needs for software changes due to one change elsewhere in the program e.g. the change in the view necessitated by changing a model. The paper defining ripples is from 1978, and you cite another paper about measuring ripples (2001). However to my knowledge, in agile development, refactoring (improving code quality without changing its semantic meaning) is seen as an effort that accompanies development, with a view on minimizing ripples. Unit, integration, acceptance tests are automated as far as possible so they can be run after almost every change. Ongoing refactoring without being able to test easily if the program still works is admittedly very hard. But tests well done can help with this. I think it would be useful for the reader if you would outline how ripples are relevant in XP or Scrum.

The fact that user interaction is not written into the Scrum process does not mean that it's not on the map. It is implicit in the requirements given by the users and users being part of the Agile team. Typically, users that are part of the Agile team will accept or not the software created. User tests can be part of the acceptance procedure, usability improvements can be features in a Scrum process.

While user experience is put to the forefront, the paper is quite sparse on the processes and on citations regarding HCI.

You state: " *Unfortunately HCI is the most ignored and unattended phase of scientific software solution development*". To my impression, this is not the state of the art any more, and the EBI even have specialized UX personnel who can be called into projects.

The authors then describe what they see as reasons for poor HCI properties of scientific software and state that no-one uses software with bad HCI and state that software with bad HCI won't be used. In my (anecdotal) experience, many software products start out as concrete problem solvers for their authors, with bad HCI (for others, as the original author writes the software tuned to his/her own needs) which then are successively refined to serve a larger public. Of course this process bears its problems for outside users. However, such software gets adopted, because of word of mouth.

In the article, HCI "design patterns" are not laid out in sufficient detail. The normal forms (1NF) etc. could be either treated more shortly or in more detail. The enumerating of 1NF to 5NF and not saying what they signify is in my view not the right middle ground.

---

" *Real time examples*" should be changed to "Real life examples"

I think these examples are an interesting overview, but to my feeling they are not addressing what is needed to support the claims made about the *Butterfly *process. Rather than giving a list of software artifacts that were created using the process I would prefer more information about the outcome of the different phases. What were iterative improvements etc.? If you are using a ticketing systems and a versioning system, it is highly likely that you can generate from logs a compelling story that lets the readers know more about the *Butterfly *process in everyday life. In particular it would be interesting to learn more about how many people performed the processes, and how the roles were distributed. 

Such an approach would also benefit the software system comparison of systems that did not use *Butterfly*. Following the *Butterfly *paradigm will take time and effort. Was a sufficient amount of time available for the systems used for the overview?

---

All in all, I find this paper is a mixture between an opinion piece, a methods piece, and an overview piece. Even if I do not agree with many claims made in the paper, I do think the question of whether scientific software engineering (or even some scientific domains) need special software engineering processes is an interesting one (albeit maybe not a new one).

I have read this submission. I believe that I have an appropriate level of expertise to confirm that it is of an acceptable scientific standard, however I have significant reservations, as outlined above.

# Response

**Published:** 2014-07-27

**Authors:** Zeeshan Ahmed

**Affiliations:** Julius-Maximilians-University Wurzburg, Germany

**Thank you so much for your time in reviewing our manuscript and giving valuable suggestions, as we believe that following those our manuscript has been improve a level ahead.**

Referee Comment:

*"Caveat: I lead a group that does scientific software development. I am interested in software development both from the conceptual and the technical side. However, I would not be a serious contender for a software engineering chair. As a consequence, I cannot claim full knowledge about the state of the art of software engineering processes.*

*I agree to the following takeaways that I pull out of the paper:*


*     *

- *If you know you are building an application which is for other people than yourself, it makes sense to follow a software engineering process.*
- *Think about maintainability and sustainability of your software.*
- *Think about your users and write usable software.*
- *Operate using cyclic refinement*

**We agree with you.**

Referee Comment:

*"The Butterfly model is an assembly of current software practices, emphasizing the needs of the scientific software developer. I see the strength of the article in giving a concise overview of what a scientific software developer should do."*

**Thanks, and we agree with you.**

Referee Comment:

*"However, I disagree with some statements made in the paper. Some of these statements relate rather to anecdotal experience. This points to one of the weaknesses in the paper: There are statements made with respect to the scientific software domain that are not marked as anecdotal, but which are not backed by either empirical facts (e.g. questionnaires) or pointers to such facts in the literature."*

**Thank you for pointing this out - we respect your opinion.**

Referee Comment:

*"In more detail:*

*Generally I agree, science is a fast-paced environment with rapidly changing requirements asking for suitable software engineering processes."*

**True.**

Referee Comment:

*"Current software engineering and development*

*The paper comprises a large number of citations. However, I do not agree with the insights taken by the authors from that literature."*

**Thanks, and we respect your opinion.**

Referee Comment:

*"To my knowledge, the field of Software Engineering has realized that one key factors for success is if software matches the needs of users."*

**True.**

 Referee Comment:

*"Agile Development follows the view that often-times, users don't even know what they need until they are closely involved in development and see where their own ideas of their work (i.e. using the program) could fail. This is why there is less of an initial requirements analysis, but there is a series of sprints that each seek the implementation of features. Each feature implemented also influences the next stage of the requirements analysis.*"

**We agree with you.**

Referee Comment:

*"The authors of this paper see the lack of quality software production as one of the weaknesses of agile software engineering practices. However, Agile software engineering practices like XP and Scrum emphasize the importance of testing and other practices intended to increase the software quality. Such processes definitely do not disregard the production of quality production. So, to cut a long story short, I do not agree with the impression created in the paper that agile processes lead to muddle-through software that is unstable and short term due to the process."*

**We respect your opinion and want to explain here that the aim of this paper is not at all to present Agile software engineering practices as muddle-through approaches, but to state that the “ *Butterfly”*** ***paradigm*** **is more useful in some specific scenarios. Our aim is to generalize the basic software engineering concepts and stream line them in a way that most of the people in Bioinformatics (non-informatics or with more biology background) can easily adopt them. Hence, Agile software engineering practice is of course a viable approach, and we have revised our paper and tried to elaborate the proposed new approach in more detail, by giving a case study of a real time scientific solution’s development and being careful not to misrepresent alternatives.**

Referee Comment:

*"You criticize the "ripples" through the program that come from changes. Ripples is the word describing the needs for software changes due to one change elsewhere in the program e.g. the change in the view necessitated by changing a model. The paper defining ripples is from 1978, and you cite another paper about measuring ripples (2001). However to my knowledge, in agile development, refactoring (improving code quality without changing its semantic meaning) is seen as an effort that accompanies development, with a view on minimizing ripples."*

**Thanks for addressing “ripples”. We respect your opinion and you’re points are valid, in particular regarding Agile development. However, our point is summarised here (expanded on in revised text):**

**Adding new features to existing applications on a random basis in academia, can cause unidentified and illogical errors to occur which can badly effect the stability of the application.**

**For example - we have implemented some operations with mutual consent of all the team members (including scientists, students, lab operators and computer scientists etc.) and then when the application’s new version is completed, a new change comes from the team. This requires the selective removal of implemented options with the addition of new connecting features, giving a high probability that errors will occur.**

Referee Comment:

*"Unit, integration, acceptance tests are automated as far as possible so they can be run after almost every change. Ongoing refactoring without being able to test easily if the program still works is admittedly very hard. But tests well done can help with this. I think it would be useful for the reader if you would outline how ripples are relevant in XP or Scrum."*

**We respect your opinion and you are absolutely right. So we now touch upon the questions of ripples regarding XP and scrum. In our personal academic experience we have noticed that especially in those cases when the developer is not the user of the real time scientific software application (which will be used in the laboratories or in fields e.g. our developed solutions DroLIGHT or Ant-App-DB), it is really hard to perform extensive on-time testing (including Unit, integration, acceptance tests etc.).**

**One cannot receive the greatest benefit from testing in these situations. So when** **a developer has to keep randomly and extensively changing things in an application without on-time testing then there is a high probability of getting ripple effects.**

Referee Comment:

*"The fact that user interaction is not written into the Scrum process does not mean that it's not on the map. It is implicit in the requirements given by the users and users being part of the Agile team. Typically, users that are part of the Agile team will accept or not the software created. User tests can be part of the acceptance procedure, usability improvements can be features in a Scrum process. While user experience is put to the forefront, the paper is quite sparse on the processes and on citations regarding HCI.*

*You state: "Unfortunately HCI is the most ignored and unattended phase of scientific software solution development". To my impression, this is not the state of the art any more, and the EBI even have specialized UX personnel who can be called into projects."*

**You are absolutely right and we agree with you but this is more applicable when a commercial application is developed or some specialised software engineering or related group is producing the solution. We added HCI citations and mention the EBI approach as an example of recent trends. Furthermore, it is true, HCI is extensively ignored when a new scientific software solutions is developed in most of the scientific academia.**

**Along with the heavy scientific software solution development, we are also taking part in analysing different scientific software applications for different purposes e.g.**


** **

- **Dandekar T, Fieselmann A, Saman M, and Zeeshan Ahmed. "** **Software Applications toward Quantitative Metabolic Flux Analysis and Visualization** **", Briefings in Bioinformatics, Oxford University Press, Oxford Journals; 15(1): 91-107, 2014.**
- **Zeeshan Ahmed, Saman M, Dandekar T:** **Computational feature performance and domain specific architecture evaluation of software applications towards metabolic flux analysis** **.  Recent Patents on Computer Science, 5(3):165-176, 2012.**

** **


**We have analysed and used many different developed software applications (more than 35) and scripts. With reference to our published work, most of the time is consumed in configuring the applications and understanding its features. If a person with an Informatics background cannot easily configure and use Bioinformatics solutions then how can a layman.**

**Moreover, when it comes to follow some already developed open source application, and add some features to it - then if the pre-processed source code is quite large and without any formal documentation, it can be a nightmare for Bioinformatics students.**

Referee Comment:

*"The authors then describe what they see as reasons for poor HCI properties of scientific software and state that no-one uses software with bad HCI and state that software with bad HCI won't be used. In my (anecdotal) experience, many software products start out as concrete problem solvers for their authors, with bad HCI (for others, as the original author writes the software tuned to his/her own needs) which then are successively refined to serve a larger public. Of course this process bears its problems for outside users. However, such software gets adopted, because of word of mouth."*

**We respect your opinion and agree with you, and have altered the statements accordingly the revision.**

Referee Comment:

*"In the article, HCI "design patterns" are not laid out in sufficient detail."*

**Following your suggestion, we have revised it and have added some information about HCI design patterns.**

Referee Comment:

*"The normal forms (1NF) etc. could be either treated more shortly or in more detail. The enumerating of 1NF to 5NF and not saying what they signify is in my view not the right middle ground."*

**Thanks for the opinion and following your suggestion, we have revised it.**

****

**Here our point was not to elaborate on database normalisation forms but emphasise its use. In our previous experience (without denigrating any researcher or developer), we have noticed that often, databases are created to store and manage data - but there are no relationships between entities. This can have a negative impact - especially in search and indexing operations. Moreover if data is well normalized then for large datasets, it will expedite the processing speed in searching the elements.**

Referee Comment:

*"Real time examples" should be changed to "Real life examples"*

**Following your suggestion, we have revised the heading and the examples have been augmented.**

Referee Comment:

*"I think these examples are an interesting overview, but to my feeling they are not addressing what is needed to support the claims made about the Butterfly process. Rather than giving a list of software artifacts that were created using the process I would prefer more information about the outcome of the different phases. What were iterative improvements etc.? If you are using a ticketing systems and a versioning system, it is highly likely that you can generate from logs a compelling story that lets the readers know more about the Butterfly process in everyday life. In particular it would be interesting to learn more about how many people performed the processes, and how the roles were distributed."*

**We agree with you.**

****

**We have revised our paper and tried to elaborate the proposed approach in more detail, by giving a case study of a real time scientific solution’s development. Questions such as iterative improvement are answered by concrete examples.**

Referee Comment:

*"Such an approach would also benefit the software system comparison of systems that did not use Butterfly. Following the Butterfly paradigm will take time and effort. Was a sufficient amount of time available for the systems used for the overview?"*

**You are absolutely right here that there must be some comparison drawn between the software applications developed using Butterfly and without. The problem here is that our proposed paradigm is quite new and this paper is the first public, methodological debate on it. Moreover according to the scope of this manuscript, we have only presented our approach with some examples but in future we will definitely try to come up with more intensive comparisons. We are still working on the strengthening our approach. In particular, for sustainable development of an application the Butterfly paradigm did boost results - for instance developing metabolic modelling software (LS-MIDA, Isotopo, LipidPRO including database applications and data management) or neurobiology software (the running example of the paper, the DroLIGHT series of programs). We now present the approach to others and note that we also have good results in developing the first version of the App-Ant database, an application oriented behaviour monitoring system, again with a planned longer term pipeline development.**

Referee Comment:

*"--- All in all, I find this paper is a mixture between an opinion piece, a methods piece, and an overview piece. Even if I do not agree with many claims made in the paper, I do think the question of whether scientific software engineering (or even some scientific domains) need special software engineering processes is an interesting one (albeit maybe not a new one)."*

**Thank you so much for your valuable time in reading and evaluating our work and giving your opinions to further improve it. We have tried to follow all your helpful suggestions and those which are still left partly open due to the scope or limits of this paper will soon be answered by further efforts. We have strengthened the methods section (see also our response to Paul Vauterin) but kept our opinion and own approach as well as added more citations for an overview.**

# Peer review: Referee response for version 1

**DOI:** 10.5256/f1000research.3945.r4632

**Published:** 2014-05-12

**Authors:** Paul Vauterin

**Affiliations:** 1 Wellcome Trust Centre for Human Genetics, University of Oxford, Oxford, UK

This paper addresses some major and often overlooked problems associated with software development in an academic environment:

- Lack of long-term vision due to short contract cycles.
- Lack of attention to usability aspects such as well-thought user interfaces.
- Lack of documentation, training material, and other follow-up.

I share the opinion of the authors that these elements pose a substantial threat to the quality of the scientific software products that are created in academic environment. Lots of valuable academic software development projects fail because of one or more of these reasons, resulting in a waste of efforts. Therefore, I think that papers focusing on solutions to overcome these issues certainly deserve a place.

A substantial portion of the paper reads like an introduction to standard software engineering practices, with a focus on scientific software development.  It is generally well written, and does provide a useful resource for academic software developers who are interested in learning more about this subject.

In addition, the authors present the “Butterfly model”, which they claim is a novel approach to software engineering, specifically tailored towards scientific software development. It does contain some interesting aspects, specifically the integrated attention to Human-Computer-Interaction (HCI), something often vastly overlooked in academic software development. However, in my opinion the authors tend to overstate the originality of this approach, as it mostly recuperates already known concepts and practices. It does have the merit that it attempts to capture these concepts in a single framework focusing on scientific software development in an academic environment.

Perhaps the major problem I have with the way this model is presented is that the authors do not clearly mention its scope of application. Clearly, this model only makes sense for larger and longer-term development projects, whereas lots of academic (bioinformatics) software projects are very small, simple in structure, and short-lived by nature. Applying the “Butterfly model” or a similar rather heavyweight approach to these projects could be a counter-productive and frustrating. In my opinion, the authors missed the opportunity to stratify the preferred approach according to the size and complexity of the project at hand. A consequent risk is that a reader might be confused and wrongly informed, thinking that this approach should be used for every software development effort, regardless its size. I think that the authors should properly address the scope of the model in the paper.

The authors provide a rather large set of software products they claim were developed with the Butterfly model, but do not provide much detail about what this meant in practice. I believe that the paper would vastly benefit if one specific case was picked, for which the practical approach of the Butterfly model would be elaborated, and the benefits would be highlighted in a very concrete way. This would also help to substantiate the author's claim about the novelty of the Butterfly model.

Further comments:

 

- *“Moreover the SDLCs famous for the quick development (Rapid Prototype Model, Agile Development Model, Extreme Programming Model, Evolutionary Model, Code and Fix Model), lack in quality software production*”:
  This is a rather controversial statement, as the list includes some generally respected models. Since the paper does not provide further arguments to back this claim, I believe it should not be made.
- *“Here, function requirements are those which can be implemented (based on existing resources, time, budget, labor, tools, technologies and methodologies), and non-functional requirements are those which cannot be implemented”*:
  I do not believe that this the standard definition of function(al) vs. non-functional requirements. Functional requirements specify what the system should do (e.g. input-behaviour-output), whereas non-functional requirements specify how it should do that (e.g. qualities such as speed, robustness, scalability, maintainability, …). 
- *“Real time examples using Butterfly”: *
  I think this should read “Real **life** examples…”. Other ways, I can’t interpret this title.

 

- The link to the “ *Isotopo software*” ( http://www.tr34.uni-wuerzburg.de/computations/isotopo/) was broken at the time this report was written. In an ironic way, this seems to illustrate one of the points of the authors about the short-livedness of many academic software projects.

 

- I believe that this article should be catalogued as “Methods Article” rather than “Research article”.

I have read this submission. I believe that I have an appropriate level of expertise to confirm that it is of an acceptable scientific standard, however I have significant reservations, as outlined above.

# Response

**Published:** 2014-07-27

**Authors:** Zeeshan Ahmed

**Affiliations:** Julius-Maximilians-University Wurzburg, Germany

**Thank you so much for your time in reviewing our manuscript and giving valuable suggestions, which all helped to further improve our manuscript.**

Referee Comment:

*"This paper addresses some major and often overlooked problems associated with software development in an academic environment:*

- *Lack of long-term vision due to short contract cycles.*
- *Lack of attention to usability aspects such as well-thought user interfaces.*
- *Lack of documentation, training material, and other follow-up."*

 

**We agree with you.**

 Referee Comment:

*"I share the opinion of the authors that these elements pose a substantial threat to the quality of the scientific software products that are created in academic environment. Lots of valuable academic software development projects fail because of one or more of these reasons, resulting in a waste of efforts. Therefore, I think that papers focusing on solutions to overcome these issues certainly deserve a place."*

**Thanks.**

 Referee Comment:

*"A substantial portion of the paper reads like an introduction to standard software engineering practices, with a focus on scientific software development.  It is generally well written, and does provide a useful resource for academic software developers who are interested in learning more about this subject."*

**We agree with you, and thanks for appreciating these points.**

Referee Comment:

*"In addition, the authors present the “Butterfly model”, which they claim is a novel approach to software engineering, specifically tailored towards scientific software development. It does contain some interesting aspects, specifically the integrated attention to Human-Computer-Interaction (HCI), something often vastly overlooked in academic software development. However, in my opinion the authors tend to overstate the originality of this approach, as it mostly recuperates already known concepts and practices. It does have the merit that it attempts to capture these concepts in a single framework focusing on scientific software development in an academic environment."*

**We agree with you, stressing important points of our paper.**

Referee Comment:

*"Perhaps the major problem I have with the way this model is presented is that the authors do not clearly mention its scope of application. Clearly, this model only makes sense for larger and longer-term development projects, whereas lots of academic (bioinformatics) software projects are very small, simple in structure, and short-lived by nature. Applying the “Butterfly model” or a similar rather heavyweight approach to these projects could be counter-productive and frustrating. In my opinion, the authors missed the opportunity to stratify the preferred approach according to the size and complexity of the project at hand. A consequent risk is that a reader might be confused and wrongly informed, thinking that this approach should be used for every software development effort, regardless its size. I think that the authors should properly address the scope of the model in the paper.*

*The authors provide a rather large set of software products they claim were developed with the Butterfly model, but do not provide much detail about what this meant in practice. I believe that the paper would vastly benefit if one specific case was picked, for which the practical approach of the Butterfly model would be elaborated, and the benefits would be highlighted in a very concrete way. This would also help to substantiate the author's claim about the novelty of the Butterfly model."*

**Thanks for these well taken suggestions - we agree with you, and tried to incorporate these. We have heavily revised the manuscript and now, along with some previously given examples, we have added a case study. Each layer of the** ***Butterfly*** **model is now explained with this example of its implementation, explaining the way we have applied the concepts in a real time scientific software solution’s development. We hope this can clarify most of the ambiguous or unclear points including getting an idea on the specific merits and originality as well as the stratification of the approach, e.g. how much effort should be devoted at each step, looking at the concrete examples given.**

Referee Comment:

*"Further comments: “Moreover the SDLCs famous for the quick development (Rapid Prototype Model, Agile Development Model, Extreme Programming Model, Evolutionary Model, Code and Fix Model), lack in quality software production”:*

*This is a rather controversial statement, as the list includes some generally respected models. Since the paper does not provide further arguments to back this claim, I believe it should not be made."*

**Following your suggestion, we have revised this paragraph accordingly.** 

Referee Comment:

*"“Here, function requirements are those which can be implemented (based on existing resources, time, budget, labor, tools, technologies and methodologies), and non-functional requirements are those which cannot be implemented”:*

*I do not believe that this the standard definition of function(al) vs. non-functional requirements. Functional requirements specify what the system should do (e.g. input-behaviour-output), whereas non-functional requirements specify how it should do that (e.g. qualities such as speed, robustness, scalability, maintainability)."*

**We agree and thank you for the clarification, the paragraph has been corrected accordingly.**

Referee Comment:

*“Real time examples using Butterfly”:* 

*I think this should read “Real* ***life*** *examples…”. Other ways, I can’t interpret this title.*

**Following your suggestion, we have revised this sentence.**

Referee Comment:

*"The link to the “Isotopo software” (* *http://www.tr34.uni-wuerzburg.de/computations/isotopo/* *) was broken at the time this report was written. In an ironic way, this seems to illustrate one of the points of the authors about the short-livedness of many academic software projects.*

**We have revised and updated the web link.**

Referee Comment:

*"I believe that this article should be catalogued as “Methods Article” rather than “Research article”."*

**We agree with you, and we changed the category of the manuscript.**

**Thank you so much for your valuable time in reading and evaluating our work, giving your advice to further improve it. We tried to follow your suggestions as much as possible in the revision. Furthermore, all those questions which are still incompletely resolved due to the scope or limitations of this paper, we will work on further in the future.**
